# Injection of air to facilitate amniotic membrane preparation

**DOI:** 10.5935/0004-2749.202100109

**Published:** 2021

**Authors:** Marcelo Bezerra Diógenes, Felipe Nunes de Miranda, Dácio Carvalho Costa

**Affiliations:** 1 Hospital Geral de Fortaleza, Fortaleza, CE, Brazil

Dear Editor,

The amniotic membrane is increasingly used in ophthalmology^(1)^. It is now regularly used as an alternative to established treatments of ocular surface disorders to protect and shield the eye from further degeneration, support damaged tissue, and promote re-cellularization^(2,3)^. Disorders that can be treated by amniotic membrane transplantation include limbal stem cell def iciency, bullous keratopathy, corneal ulcer, descemetocele, corneal perforation, complications of glaucoma surgery, symblepharon, pterygium, and tumor resections^(2,4)^. The benefits are derived from biological features inherent to the membrane, including anti-inflammatory, antimicrobial, and antiangiogenic activity and a lack of immunogenicity^(4,5)^.

Placental tissue is widely available, inasmuch as it is expelled during birth^(1,2)^. Unfortunately, amniotic membrane is not as accessible to surgical use, probably because the processing of this material is a laborious and delicate procedure for which medical staff must be trained^(1)^. Our aim was to simplify and expedite amniotic membrane preparation through a simple, reproducible air injection technique.

**Figure 1:**
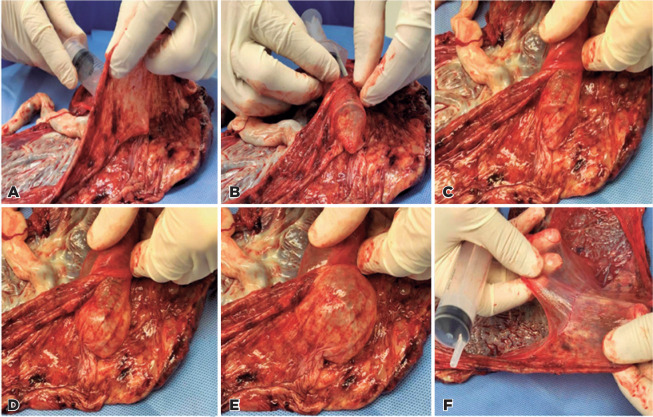
(A, B, C) The introduction of a 20-mL syringe and injection of air between the two membranes. (D, E) Use of the left hand to seal the incision cut. (F) Separation of the amnion from the chorion.

**Figure 2:**
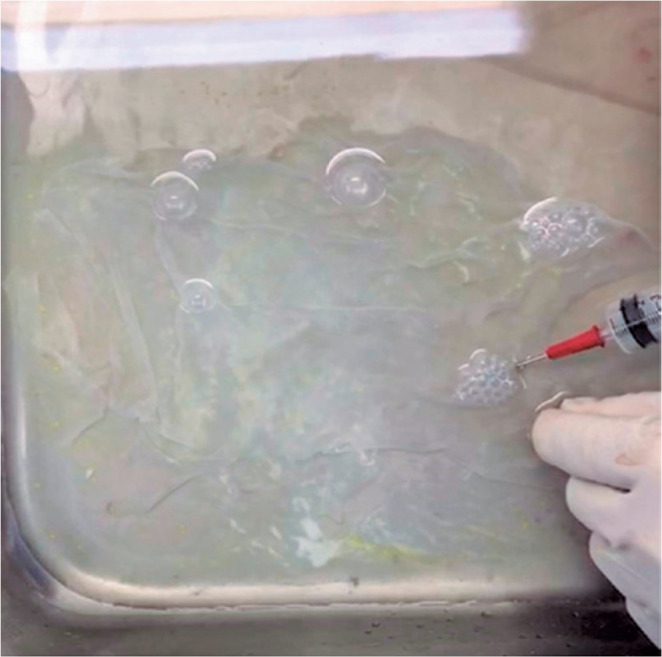
To ensure that the epithelial side of the membrane faces up, we injected air beneath the floating membrane.

Donors were seronegative for HIV, syphilis, and hepatitis B and C viruses. The placentae were collected after elective cesarean delivery with the written consent of each donor^(4)^. Thirty minutes after delivery, each membrane was processed under sterile conditions by a first-year ophthalmology resident under supervision, as follows: Initially, the placenta was washed with 0.9% saline solution^(3)^. A cut was then made adjacent to the umbilical cord in order to dissect the amnion from the chorion with a blunt scissor^(1)^. When the dissected area was big enough, we introduced a 20-mL syringe between the two membranes and injected air with one hand, using the other hand to seal the incision cut (Figure 1). This procedure was repeated to fully separate the tissues. The amniotic membrane was retrieved in two large pieces, which were washed thoroughly with saline solution to remove blood clots^(1)^. Then the membranes were kept for 10 minutes with the epithelial side up in a solution containing 1000 mL of 0,9% saline, 1200 UI of penicillin, 50 mg of amphotericin B, and 100 mg of amikacin^(2,5)^. To ensure that the epithelial side was up, we injected air beneath the floating membrane (Figure 2). We subsequently stretched the amniotic membranes onto nitrocellulose paper and cut it into pieces measuring 3 x 3 cm^3^. The pieces were then stored in a sterile container with glycerol for preservation^(4)^.

Our technique for amniotic membrane preparation is simple and reproducible; the air can be injected with a 20-mL syringe. With the air injection, the amniotic membrane could be retrieved by a nonexperienced clinician in two large pieces, without loss of tissue. Also, we noticed that the dissection was faster with the new technique.

The amniotic membrane is the avascular, semitransparent, innermost portion of the placenta^(1)^. It is the most delicate of the fetal membranes, with a thickness ranging from 0.02 to 0.5 mm^(1,4)^. At approximately the 12th week of gestation, the spongy layer of the amniotic sac fuses with the chorion, and they remain fused throughout gestation^(4)^. During the separation of the fetal membranes after birth, these structural aspects have to be taken into account. Therefore, the tissue preparation must be a long and delicate procedure performed by trained personnel^(1)^; otherwise, damage can result, and good tissue can be lost.

Our idea came from the big bubble technique for lamellar transplantation, a less traumatic approach to separate tissues. Using air injection, we were able to separate the membranes with less manipulation and friction of the amnion’s epithelial side. The integrity of the epithelial side is essential for the use of its antiangiogenic and anti-inflammatory properties as a biological bandage^(4)^.

In addition, no tears or loss of tissue occurred during preparation, and thus we were able to retrieve two big pieces of amniotic membrane. This facilitated the final cut of the membrane into 3 x 3 cm pieces. We believe that big pieces of amnion can be of great use in nonophthalmologic applications that require larger dressing bandages, such as those for nonhealing skin ulcers, burns, vaginal reconstruction, prevention of surgical adhesions, repair of abdominal herniation, and head and neck surgery^(1,2)^. Because the nonexperienced clinician could retrieve the tissue quickly and without loss, we believe this technique has a reasonable learning curve and the potential to increase the accessibility of amniotic membrane without additional cost.
